# In Search of Functional Advantages of Knots in Proteins

**DOI:** 10.1371/journal.pone.0165986

**Published:** 2016-11-02

**Authors:** Pawel Dabrowski-Tumanski, Andrzej Stasiak, Joanna I. Sulkowska

**Affiliations:** 1 Centre of New Technologies, Banacha 2c, 02–097, Warsaw, Poland; 2 Faculty of Chemistry, University of Warsaw, Pasteura 1, 02–093, Warsaw, Poland; 3 Center for Integrative Genomics, University of Lausanne, 1015-Lausanne, Switzerland; 4 SIB Swiss Institute of Bioinformatics, 1015-Lausanne, Switzerland; Russian Academy of Medical Sciences, RUSSIAN FEDERATION

## Abstract

We analysed the structure of deeply knotted proteins representing three unrelated families of knotted proteins. We looked at the correlation between positions of knotted cores in these proteins and such local structural characteristics as the number of intra-chain contacts, structural stability and solvent accessibility. We observed that the knotted cores and especially their borders showed strong enrichment in the number of contacts. These regions showed also increased thermal stability, whereas their solvent accessibility was decreased. Interestingly, the active sites within these knotted proteins preferentially located in the regions with increased number of contacts that also have increased thermal stability and decreased solvent accessibility. Our results suggest that knotting of polypeptide chains provides a favourable environment for the active sites observed in knotted proteins. Some knotted proteins have homologues without a knot. Interestingly, these unknotted homologues form local entanglements that retain structural characteristics of the knotted cores.

## Introduction

Proteins belonging to several unrelated protein families fold towards their native structures in such a way that their polypeptide chains get tied into knots [1–3]. Most of them form simple trefoil knots, but there are also proteins forming more complex knots such as figure-of-eight, pretzel-like pentaknot and Stevedore’s knot [1, 4–6]. It should be stressed here that these knots are not results of some accidental entanglements of long polypeptide chains but their complex structure and topology is entirely dictated by their sequence [7, 8]. However, folding of knotted proteins is much slower and thus less efficient than of unknotted analogues [7–9]. In that respect, the requirement to form a knot during folding provides evolutionary disadvantage. Yet, several known families of knotted proteins have their knotted domains strongly conserved even in lines of organisms that got separated more than over a billion years ago [10].

We investigate here what can be the structural and functional advantage of tightly knotted protein folds that have made them conserved during evolution. We specifically check whether portions of polypeptide chains directly involved in knot formation have gained some special properties. We observed that amino acids positioned at the borders of knotted cores show strongly increased number of contacts giving them the possibility to form various chemical bonds. Borders of the knotted cores showed also increased thermal stability and decreased solvent accessibility. We also look more closely at how the knotted core contributes to the formation of catalytic centres and binding sites for substrates and cofactors. In addition, we compare structural properties of two very similar in structure and function protein homologs where one is knotted and the other unknotted.

## Results

**Fig 1:**
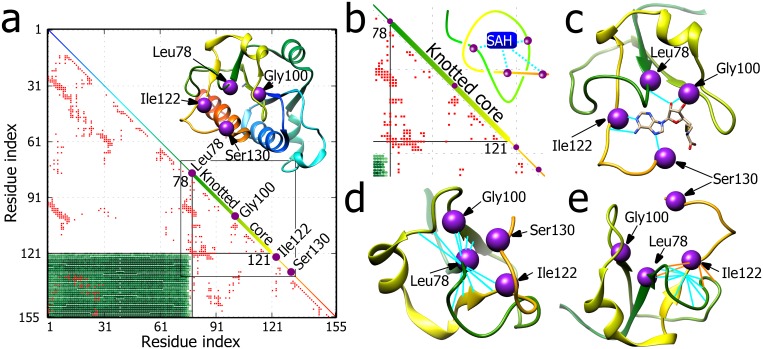
Knotted core and active sites in TrmL protein. **a.** Structure of TrmL protein (PDB id 4JAK, chain A of the dimer) is shown with a matrix encoding the knot type of every subchain (see the main text). The diagonal shows the linear map of the chain. Colours permit finding corresponding regions in the structure shown above the diagonal. The knotted core is indicated along the diagonal as a bold line. Violet beads placed along the protein chain and the diagonal indicate the positions of four amino acids binding SAH. The red entries in the matrix denote intra-chain contacts. Black lines mark the knot termini. The numbers denote residues’ indices. **b.** An enlarged portion of the matrix with simplified representation of the core forming right-handed 3_1_ knot. Violet beads indicate the positions of amino acids participating in the SAH binding (cyan lines). **c.** SAH bound to the “highlighted” residues within the knotted core. **d.** Leu78, located in the knotted core entry and which is one of the amino acids contributing to SAH binding site, shows large number of contacts connecting it with the encircling polypeptide chain. **e**. Ile122, which is located immediately after the exit point from the knotted core also shows a large number of contacts with the encircling polypeptide chain (orange stripes). This number is not as high though as that of the preceding amino acid that is located at the exit point from the core (cyan stripes).

In our search of functional advantages of knots, we concentrate on proteins forming trefoil knots as these are the most frequently observed among knotted proteins. Analysis of the geometry of protein knots leads to the definition of knotted core and knot tails [10–12]. The knotted core is the smallest subchain of the entire polypeptide chain, which still forms the knot, while the tails are the parts of the chain remaining on both sides of the knotted core (see Fig 1). Searching for possible functional advantages of knots in proteins, we focus on deeply knotted proteins, since these are especially difficult to fold [13] and hence their formation my result in possibly unique properties. As deeply knotted proteins we consider those, in which the shortest knot tail is at least 20 amino acids long. In addition, we concentrate on tight knots. Since the smallest knotted core found in KnotProt database [3] consists of 42 amino acids, we set a limit of 84 amino acids (twice the minimal size) on the core size. Finally, to increase the chance of finding structural characteristics caused by knotting and not by some possible structural similarities of compared proteins, that are unrelated to knotting, we decided to analyse only the knotted proteins which are very different from each other, when comparing their structure and function. As a result, we study here three knotted proteins: a methyltransferase from the SPOUT superfamily, N-acetyl-ornithine transcarbamoylase, and a ribonucleoprotein participating in RNA splicing. Our choice is consistent with previous classification of knotted proteins showing that there are 7 different protein families containing a 3_1_ knot, but only three of them possess a deep, tight knot [14].

### Knotted core and active sites in TrmL protein

SPOUT is a large protein superfamily of rRNA or tRNA-modifying methyltransferases [15, 16]. These proteins form dimers important for activity [17] and each monomer forms a deep trefoil knot providing binding site for the cofactor of the methyl transfer reaction—S-adenosylmethionine (SAM). For SPOUT family members it was shown that amino acids located at the border of the knotted core show high conservation [18, 19]. Relatively recently one of SPOUT members, TrmL, which is a tRNA methyltransferase from E. coli was crystallized without (PDB id 4JAK) and with (PDB id 4JAL) S-adenosylhomocysteine (SAH) [20]–by-product arising when SAM already “donated” its methyl group to tRNA. The analysis of the crystal where TrmL binds SAH allows determination of the exact amino acids involved in ligand binding. It is interesting to check, whether these are located within or in the immediate vicinity of the knotted core of the protein.

Fig 1a shows the structure of TrmL protein (to facilitate perception of the polypeptide structure only one of two monomers of crystalized TrmL dimer is shown) together with a matrix representation that encodes the knotting of all subchains of the analysed protein [11, 21]. All subchains, whose starting and ending residues, indicated along the abscissa and ordinate, respectively, fall within the green rectangle can be considered as forming an open trefoil knot. Therefore, the knotted core of TrmL starts around the Leu78 residue and ends around the Thr121 residue, as indicated in the KnotProt database [3]. The extent of the knotted core is depicted on the matrix diagonal that corresponds to the linear map of the entire protein. It should be mentioned though that defining the borders of the knotted core is somewhat problematic as the borders can vary depending on the algorithm used to detect and define knots [22–24]. The main source of variance between different algorithms is the chosen closing procedure needed to characterize the knot type. Our probabilistic algorithm, implemented in KnotProt database [3], averages over a large number of randomly chosen closing directions and by this is independent of the arbitrary choice of the chain closure. In Fig 1 the violet beads placed on the structure of TrmL protein indicate positions of 4 residues that according to PDB entry 4JAL bind SAH. The positions of these 4 residues are also placed along the diagonal of the matrix. It is visible that two of these 4 residues locate at the extremities of the knotted core, with the next one locating in the core centre. Fig 1b and 1c focus on the knotted core and its immediate vicinity. Fig 1b schematically shows the open 3_1_ knot formed by the corresponding portion of the TrmL chain as well as the approximate positions of the 4 residues binding SAH. Fig 1c shows the actual structure of the corresponding portion of the protein together with the bound SAH molecule. The 3_1_ knot is visible, although its recognition may not be obvious since the polypeptide chain does not form a minimal crossing representation of the 3_1_ knot.

In addition to pinpointing the position of the knotted core, the matrix presented in Fig 1a indicates all intra-chain contacts defined as in [25] and marked as red squares. The non-local contacts stabilize formed knots and may also be responsible for the conservation of the knotted character of this protein during evolution. Hence, we focused on the contacts between amino acids that are distally located from each other along the polypeptide chain. Therefore, we imposed the condition that the sequential distance separating the contacts should be at least of 4 amino acids. We were especially interested in contacts between residues located within the knotted core or in its immediate vicinity. Particularly interesting are clusters of contacts that correspond to the regions where the chain enters or exits the knotted core. The horizontal and diagonal lines on the matrix formed by red marks clearly show that the terminal portions of knotted core form a large number of contacts. Fig 1d and 1e show these interactions for individual residues that are located at the N- and C-terminal end of the knotted core, respectively. It is visible that a large number of contacts realizable by individual amino acids are correlated with the particular architectonic motive of tightly knotted proteins where portions of their polypeptide chain are encircled in a nearly perpendicular way by other portions of the same chain forming the knotted core. It is striking that one of the 4 amino acids contributing to the active site of TrmL is exactly the amino acid at the entry to the knotted core and thus forming very extensive set of contacts. Moreover, the second active residue located just outside the knotted core also shows increased number of interactions, although this number is smaller than that of the neighbouring amino acid delimiting the knotted core (see Fig 1e). We would like to stress here that the algorithm that defines borders of knotted cores was described earlier using strict mathematical considerations for curves in space [10] and its detection of knots’ borders in proteins is absolutely independent of the number of contacts in these regions. In addition, the positions of the boundaries, determined with single residue resolution, are as listed in KnotProt [3]. Also other published methods of determining the borders of the knotted cores [22–24] gave very consistent results (See S1 File).

**Fig 2:**
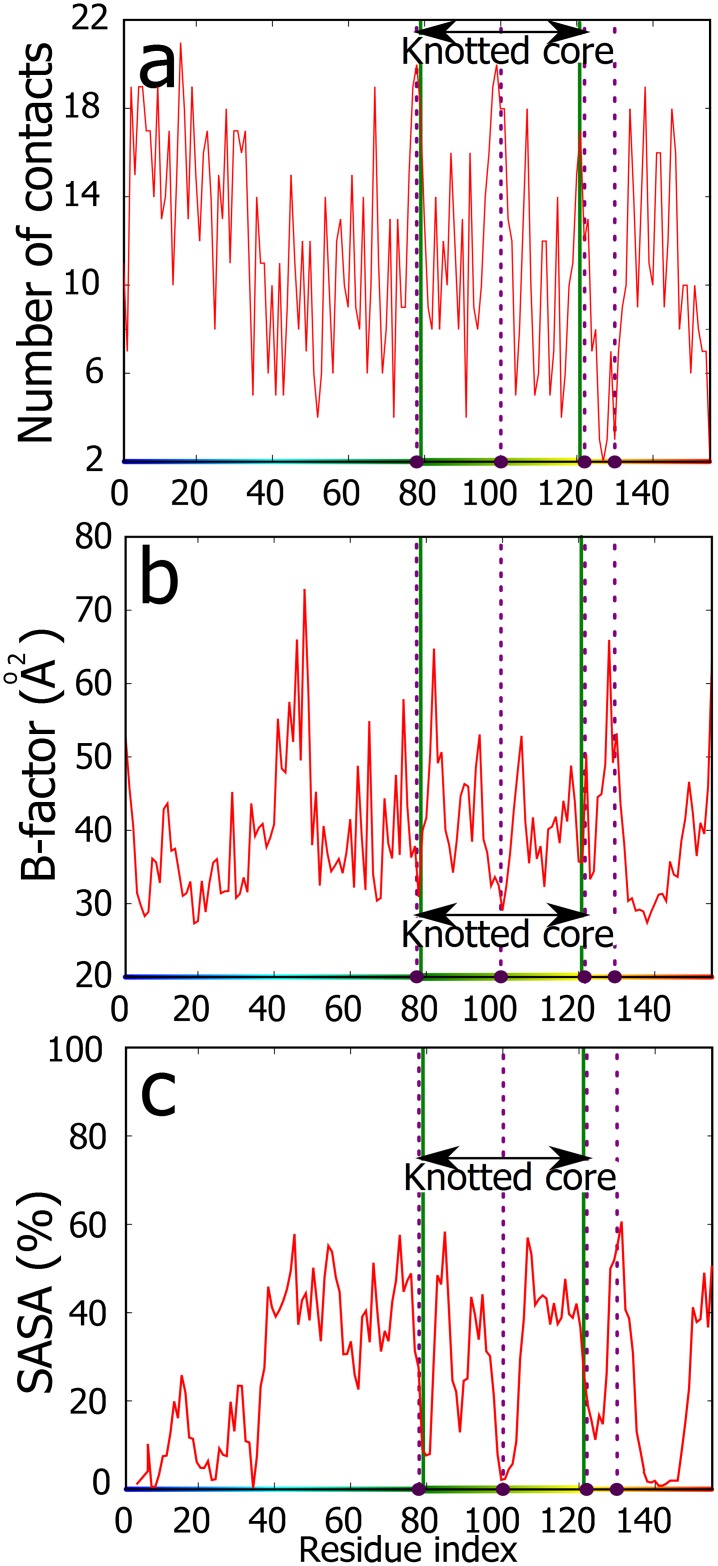
The number of contacts, relative thermal motion (B-factor) and solvent accessibility (SASA) of TrmL protein. **a.** The number of contacts in the TrmL polypeptide chain shows strong and broad maxima at the borders and in the centre of the knotted core. Out of the four amino acids involved in SAH binding the three of them locate in these three maxima of contacts. **b.** B-factor shows strong local minima at the borders and in the centre of the knotted core. Three out of four residues involved in SAH binding are located at or in the immediate vicinity of these sites. **c.** SASA shows strong local minima at the borders and in the centre of the knotted core. The SASA values are presented as running averages over a window of 8 residues. The gradient colour (horizontal axes) corresponds to colouring of the protein in the Fig 1a, the binding residues are marked by violet beads and dashed lines, and the knot core is delimited by green lines.

The increased number of contacts involving two regions where the polypeptide chain enters and leaves the knotted core (Fig 1a, 1d and 1e), inspired us to check how the number of intra-chain contacts per residue varies along the polypeptide chain of TrmL. Fig 2a shows that both borders of the knotted core form strong local maxima in the total number of contacts formed. In addition, these maxima are broad i.e. there are several sequentially close amino acids which form a large number of contacts. A similar high and broad maximum of contacts is also observed in the centre of the knotted core. Interestingly, each of these three broad maxima of contacts in the borders and in the centre of the knotted core “hosted” one of the four amino acids binding SAH. We should however point out, that these maxima are not strictly global. Though, when considering only short and buried long-distance contacts (i.e. excluding water mediated contacts), these maxima exceed all the others (data not shown).

Next, we investigated whether these three maxima of contacts provide increased stability to the borders as well to the centre of the knotted core as compared to the rest of the protein. Fig 2b shows the B-factor profile (the measure of atoms mobility) obtained for the crystallized protein. It is well visible that these three maxima with many contacts correspond to the regions that show increased stability. The stabilization of knotted cores was proposed earlier as a possible functional advantage of knotted proteins [1, 26–29]. Our analysis revealed though that this stabilization is strongly pronounced at the borders and the centre of the knotted core. As shown in Fig 1d and 1e these regions show many stabilizing interactions that are correlated with the fact that in tight knots there are tight clasps where one segment is locally encircled by the other. It is tempting to speculate that evolutionary conservation of knotted proteins is connected with the specific properties provided by the clasp motifs where one polypeptide chain turns around another chain in a nearly perpendicular orientation [30].

Finally, we analysed the solvent accessibility (SASA) of all residues in the functional dimeric, *apo* form of the protein (see Fig 2c). As could be intuitively expected the exit/entry regions into the knotted core form strong local minima in the solvent accessibility. Interestingly, these low accessibility regions are in the immediate vicinity of two regions that are highly accessible to the solvent and are located just outside of the knotted core. Considering now the solvent accessibility of the four amino acids that contribute to the SAH binding site we can see that the three of them that are located in the centre and in the extremities of the knotted core show very low solvent accessibility. Low solvent accessibility of these sites is required for the formation of hydrophobic pockets that are needed to bind S-adenosylmethionine [31]. On the other hand, the neighbouring regions should be solvent accessible to allow even hydrophobic solutes to approach. The fourth SAH binding amino acid is already outside of the knotted core and as such shows a high solvent accessibility, a high B-factor and a low number of contacts (see Fig 2).

Our results indicate that active sites in deeply knotted TrmL proteins locate within their knotted cores in such regions that have somewhat extreme properties caused by knotting. These properties are: large number of contacts with other parts of the chain, specific restriction of thermal fluctuation and high screening from solvent inside the knotted core. On the other hand, these residues have nearby regions that are highly exposed to solvent by being located just outside of the knotted core.

### Knotted core and active sites in AOTCases

**Fig 3:**
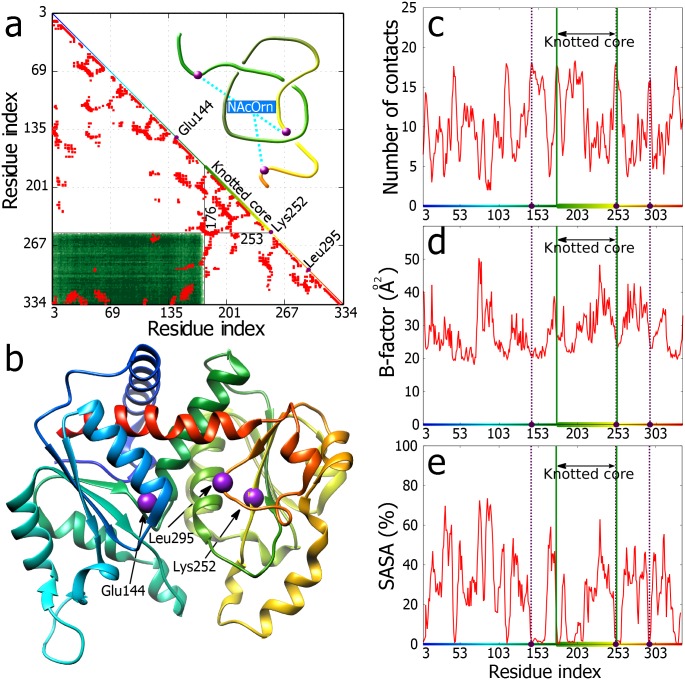
Correlation between knotting and structural characteristics of AOTCase. **a.** Matrix encoding the knotting pattern in AOTCase and intra-chain contacts (red entries). The diagonal shows the linear map of the chain and indicates placement of the knotted core. The colours correspond to Fig 3b. Black lines shows the knotted core ends with index of residues delimiting the core. In the upper triangle schematic depiction of +3_1_ knot. The violet beads represent the binding residues (cyan lines). **b.** The crystal structure of AOTCase (PDB id 3KZN) with the binding residues marked as violet beads. **c.** Total number of contacts shows local maxima for substrate binding and knot delimiting residues. One substrate-binding residue almost overlaps with the core end. Here, due to high number of residues the running average over a window of 3 residues is depicted. **d, e.** B-factor and SASA plots show increased stability and decreased solvent accessibility of the substrate-binding and knot delimiting residues. The gradient colour (horizontal axes) corresponds to colouring of the protein in the Fig 3b, the binding residues are marked by violet beads and dashed lines, and the knot core is delimited by green lines.

To check, whether other deeply knotted proteins show similar properties, we analysed N-acetyl-L-ornithine transcarbamoylase (AOTCase, PDB id 3KZN) [32], which is required for biosynthesis of arginine in several species of eubacteria. This protein was crystalized with its natural substrate acetylornithine, which permitted to determine amino acids involved in ligand binding. Fig 3a shows the matrix encoding the position of the knotted core and intra-chain contacts in the AOTCase. Along the diagonal of the matrix we indicated the location of the three amino acids involved in acetylornithine binding as well as the extent of the knotted core. It is well visible that one of the three amino acids binding acetylornithine is located at the border of the knotted core. Looking on the contact map one may again notice many contacts within the knotted core including direct interactions between the regions where the polypeptide chain enters and exits the core. Fig 3a shows a schematic presentation of the knotted portion of the protein forming a right-handed 3_1_ knot and indicates also approximate positions of the three amino acids that bind N-acetylornithine. A detailed structure of the entire protein is shown in Fig 3b.

In Fig 3c the number of contacts per residue of the AOTCase is analysed. One can see that similarly to the TrmL protein, AOTCase shows strong local maxima in the number of contacts at both borders of the knotted core. Interestingly, the three amino acids involved in acetylornithine binding are all in the regions with local maxima of contacts and one of them (as already mentioned) localizes exactly at the border of the knotted core. Fig 3d and 3e show that both the B-factor and SASA feature strong local minima at both borders and in the centre of the knotted core. Interestingly, the two other amino acids that bind acetylornithine and which are located outside of the knotted core are also in the regions with local minima of B-factor and SASA.

### Knotted core and active sites in Rds3p protein

**Fig 4:**
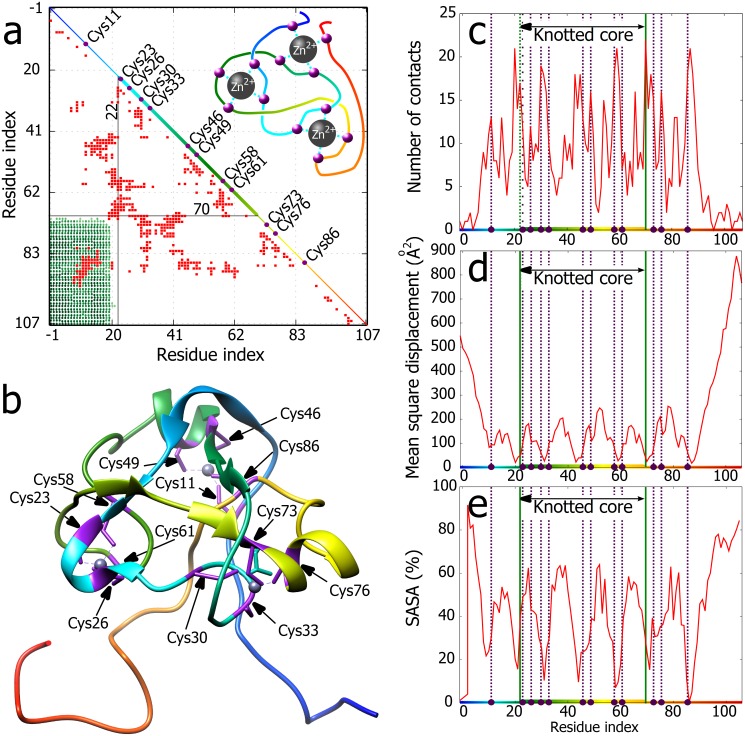
Correlation between knotting and structural characteristics of Rds3p protein. **a.** Matrix encoding the knotting pattern in Rds3p and intra-chain contacts (red squares). The diagonal shows the linear map of the chain and indicates placement of the knotted core. The colours correspond to Fig 4b. Black lines show the knotted core ends with index of residues delimiting the core. In the upper triangle schematic depiction of -3_1_ knot. The violet beads represent the zinc binding residues (cyan lines). **b.** The NMR-derived structure of Rds3p (PDB code 2K0A, model 1) with the binding residues marked in violet. The zinc ions are depicted with their interactions with cysteine residues (dashed lines). **c.** Total number of contacts shows that knot delimiting and most of binding residues are characterized by local maxima of contact number. 8 out of 12 binding residues locate inside the knotted core, two more in its exact vicinity. **d, e.** Thermal fluctuation of the residues and SASA plots show increased stability and decreased solvent accessibility of the ion-binding and knot delimiting residues. As Rds3p was analysed by NMR, instead of B-factor, the mean square displacement is depicted. The binding residues are marked by violet dots and dashed lines, and the knot core is delimited by green lines.

Our next analysed protein that forms a deep trefoil was the splicing factor Rds3p (PDB id 2K0A) characterized by NMR [33]. This protein contains 3 zinc finger motifs where each contains four cysteines involved in coordinating a zinc ion that stabilizes each domain. Fig 4a shows the matrix identifying the position of the knotted core as well as the intra-chain contacts within the Rds3p protein. It is well visible that amino acids at both borders of the knotted core show numerous contacts with other residues of the knotted core, including direct contact between the two borders. The schematic drawing in Fig 4a shows the formed left-handed 3_1_ knot together with approximate positions of 8 cysteines coordinating zinc ions. This drawing also shows that 8 out of 12 cysteines coordinating 3 zinc ions are located within the knotted core (with one immediately adjacent to the entry into the core) and two additional cysteines are located just outside of the knotted core. Fig 4b shows the detailed structure of the Rds3p protein with its 3 coordinated zinc ions.

Fig 4c, 4d and 4e show that similarly to the TrmL and the AOTCase, analysed earlier, Rds3p protein also shows strong local maxima of contacts in the regions corresponding to the two borders of the knotted core (Fig 4c) and that these two border regions form strong local minima of thermal fluctuation (Fig 4d) as well as of solvent accessibility (Fig 4e). In case of Rds3p protein the cysteines coordinating zinc ions can be considered as forming active sites of this protein. Each of three zinc ions is coordinated by four cysteines. Fig 4c, 4d and 4e show that at least two cysteines of every four localize in one of the regions with large number of contacts, small thermal fluctuations and low solvent accessibility. The majority of these regions localize within the knotted core or in the border regions of the core.

### Comparison between knotted and unknotted homologous proteins

**Fig 5:**
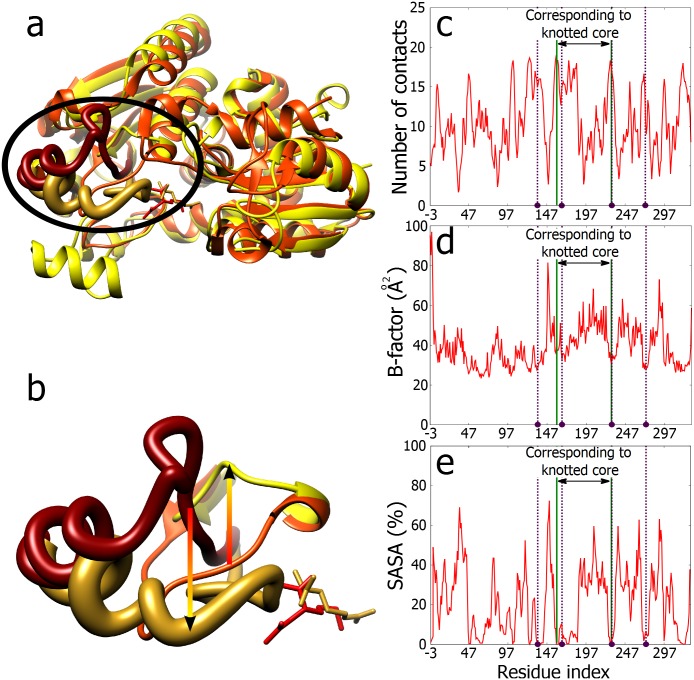
Comparison between knotted and unknotted carbamoylotransferases and structural characteristics of OTCase. **a.** Superimposed structures of knotted AOTCase (red, PDB id 3KZN) and unknotted OTCase (yellow, PDB id 4JQO) with ligands (acetylornithine and citrulline respectively, in stick representation). The thick darker fragments denote the short polypeptide chain, whose position determines the topology of the protein. The black oval shows the fragment which was enlarged in Fig 5b. **b.** Enlarged fragment of Fig 5a with topologically crucial rearrangement of the chain. In the case of OTCase chain, the (dark yellow) loop is much closer to the substrate than the analogous (dark red) loop in case of AOTCase. The gradient arrows denote the chain movement which has to be done in order to change the topology. **c.** Total number of contacts for OTCase. Here, due to high number of residues, the running average over a window of 3 residues is depicted. **d, e.** B-factor and SASA for OTCase. Binding residues are marked by violet beads and dashed lines.

We observed that in tightly knotted proteins the borders of their knotted cores are enriched in intra-chain contacts and active sites. However, formation of regions with high number of intra-chain contacts is unlikely to be limited to knotted proteins only. In fact, very similar biochemical reactions can be performed by knotted and unknotted proteins. This is the case of tRNA methyltransferases, where in addition to knotted proteins (such as TrmL protein discussed earlier) there are also unknotted proteins that show no homology to knotted methyltransferases and in which the catalytic domains form the Rossmann folds (e.g. FTSJ RNA methyltrasferase) [15]. Probably the best example of proteins with very similar structure and function, which differ with respect to knotting, is provided by two classes of transcarbamoylases: knotted AOTCases (described previously) and unknotted OTCases. These two classes of enzymes transfer carbamoyl group from carbamoylphosphate to N-acetyl-ornithine or ornithine, respectively. Both enzymes have very similar, nearly superimposable structures (Fig 5a). The crucial difference is in the vicinity of the active region where one short polypeptide section passes either on one, or another side of other short section, introducing or removing one essential crossing of the trefoil knot (Fig 5b). That local rearrangement does not change significantly the number of contacts in the relevant protein regions. Fig 5c, 5d and 5e show that substrate binding sites in unknotted OTCase with PDB code 4JQO have very similar characteristics to substrate binding sites in knotted AOTCases (see Fig 3). These sites are located in regions with large number of contacts, where thermal fluctuations and solvent accessibility are reduced. Although the change from knotted to unknotted form of transcarbamoylases did not change much the characteristics of the regions where active sites are located, one should stress that the overall structures of AOTCases and OTCases are very similar and that two out of three essential topological crossings, necessary to form the trefoil knot, are still present in the unknotted OTCase. In the place of knots in AOTCases, OTCases form entanglements that formally are not knotted though.

It is interesting to consider why the knotted and unknotted homologues differ in the substrate specificity. The presence of the knot in the AOTCase keeps one portion of the chain separated from the ligand-binding pocket and thus enlarges it (Fig 5a and 5b). This permits that pocket to bind larger cofactor—N-acetyl-ornithine. In unknotted OTCase the released chain moves towards the ligand-binding pocket (see thick chain section in Fig 5a and 5b), effectively reducing it, as would be required for binding of a smaller ligand, ornithine.

## Discussion

The analysed three examples of very different knotted proteins that are unrelated in sequence and function revealed their several similar structural properties that correlate with the presence of deep knots in these proteins. Probably the principal common property of these knotted proteins is strongly increased number of contacts in the border regions of the knotted core (see Figs 1–4). As illustrated in Fig 1d and 1e knot border regions show many contacts as there chains form tight clasps where one segment is locally encircled by the other. High number of intra-chain contacts naturally leads to high thermal stability and to exclusion of solvent from these regions. In addition to borders of the knotted core also other regions within the core show increased number of contacts with the resulting thermal stabilization and solvent exclusion (see Figs 2–4). The active sites (involved in binding of substrates, cofactors and stabilizing ions) of the three proteins show the preference to be localized in the regions with many intra-chain contacts that in turn lead to increased stability and limited solvent accessibility of these sites. Presumably, formation of tight knots with characteristic structural motifs, where portions of polypeptide chains are encircled in a nearly perpendicular direction by other portions of the same chain (see Figs 1, 3 and 4) provide very favourable environment for protein active sites. Of course, protein knotting is not necessary for the formation of regions with increased number of contacts. Knotted AOTCases and unknotted OTCases maintain very similar regions with increased number of contacts. However, unknotted OTCases do form local entanglements maintaining two out of three crossings of knotted AOTCases (see Fig 5).

## Methods

### Contact maps

The contact maps were calculated using Frustratometer server [25]. Two amino acids are treated to be in contact, if the distance between their Cβ (or Cα in case of glycine) is less then 6.5 Ǻ (short-distance contacts), or in range 6.5–9.5 Ǻ (long-distance contacts). The set of long-distance contacts splits into water mediated (between solvent exposed residues) and buried (non-exposed to solvent) [34].

### Protein structures

Protein structures were taken from RCSB database with original numbering of the residues.

### Knot detection and matrix representation

Protein topological state, matrix representation as well as knotted core and tail lengths were taken from KnotProt database [3]. For Rds3p and AOTCase proteins residue numbering had to be adjusted to RCSB numbering pattern.

### Structural comparison of the protein

The structural comparison were conducted using jFATCAT-flexible algorithm using the tool available on RCSB webpage [35, 36].

### Binding site recognition

The information about the binding site of proteins were taken directly from PDB structure on RCSB website.

### Protein depiction

Molecular graphics and analyses were performed with the UCSF Chimera package. Chimera is developed by the Resource for Biocomputing, Visualization, and Informatics at the University of California, San Francisco (supported by NIGMS P41-GM103311) [37].

### Features calculation

B-factors for TrmL and AOTCase were the mean residue values taken from the PDB structure. In case of Rds3p protein the mean square displacement of Cα atoms (recorded for each model in NMR structure) was used as an analogue of B-factor. The total SASA was computed using Chimera Software with MSMS package [37, 38]. The values obtained were normalized according to the total surface of amino acids [39] to calculate normalized SASA.

## Supporting Information

S1 File Supporting information. Comparison of methods characterizing positions of knotted cores in proteins.
(PDF)
