# Supplementary material for: In Search of Functional Advantages of Knots in Proteins
Source: PLoS One. 2016 Nov 2;11(11):e0165986. doi: 10.1371/journal.pone.0165986 (PMC5091781; doi:10.1371/journal.pone.0165986)
Supplement: S1 File — Comparison of methods characterizing positions of knotted cores in proteins. (PDF) [file pone.0165986.s001.pdf]

Supporting Information

In Search of Functional Advantages of Knots in Proteins

Pawel Dabrowski-Tumanski, Andrzej Stasiak and Joanna I. Sulkowska

Comparison of methods characterizing positions of knotted cores in proteins.

The positions of knotted cores determined using the two-point stochastic chain closure method (KnotProt) were compared to positions determined using four other chain closure techniques known from literature [1-5]. In particular, the chain was closed by means of:

1. stochastic one-point method in which the termini are extended towards one random point on a large sphere – Method 1;
2. stochastic one-direction method in which the termini are extended in one random direction and joined on the surface of a large sphere – Method 2;
3. deterministic center of the mass method in which the termini are extended from the protein's center of the mass and connected on the surface of a large sphere – Method 3;
4. deterministic center of the mass method with position of the center calculated for each analyzed subchain of the protein – Method 4.

The schemes representing each method are shown in Fig. 1. For stochastic methods we applied 1000 random chain closures for progressively shortened chains. The shortest subchain that still formed the original knot type for the majority of closures was considered as the knotted core. In deterministic methods just one closure is done for each progressively shortened chain. The shortest subchain that still forms the original knot type is considered as the knotted core. The determined positions of a knotted core according to different methods are shown in Tab. 1.

The results show that various chain-closing method used to determine the position of knotted cores in proteins give practically the same results.

Table S1 Comparison of knotted core boundaries determined by different methods. Compared are: stochastic two-point method (KnotProt default method), stochastic one-point method (Method 1), stochastic one-direction method (Method 2), deterministic center of the mass method (Method 3) and deterministic cetner of the mass method with the position of the center calculated for each subchain of the protein (Method 4). The positions were adjusted to match the PDB numbering.

PDB code	KnotProt	Method 1	Method 2	Method 3	Method 4
4JAK	78-121	78-120	78-120	77-121	79-120
3KZN	176-253	176-253	176-252	176-252	177-252
2K0A	22-70	22-68	22-69	22-71	22-71

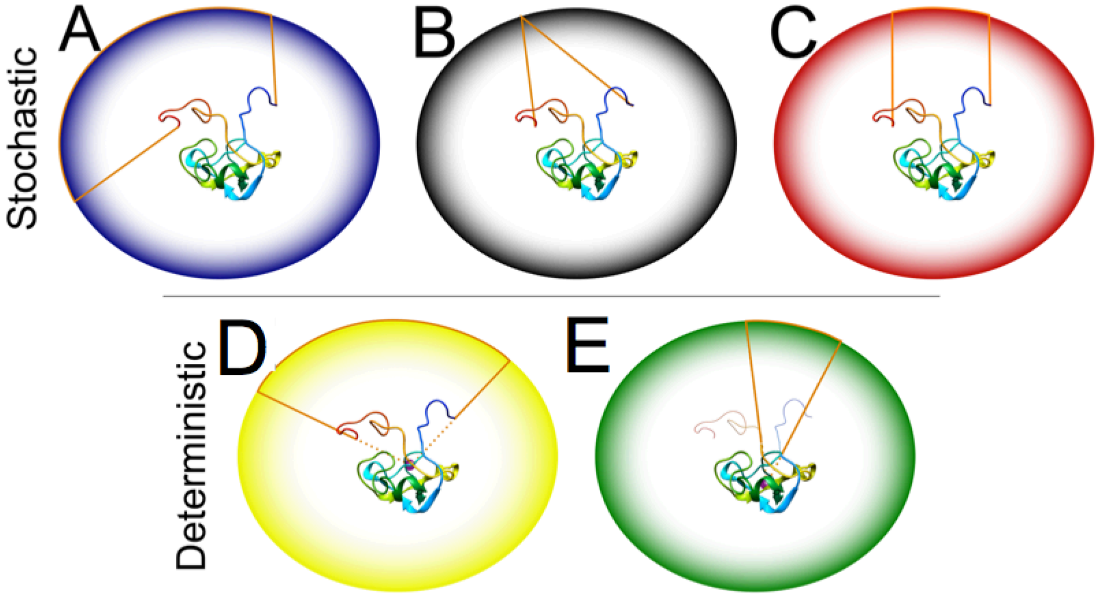

Fig. S1. Schematic presentation of closure methods used to determine the position of knotted cores in proteins: stochastic two-point method (KnotProt default method, A), stochastic one-point method (Method 1, B), stochastic one-direction method (Method 2, C), deterministic center of the mass method (Method 3, D) and deterministic center of the mass method with the position of the center of mass calculated for each subchain of the protein (Method 4, E). The orange lines denote a particular chain closure. The violet beads in D and E represent the center of mass. Note that in D the center of mass is determined for the entire protein and does not change when the chain is progressively truncated from the ends. In E the center of mass is recalculated as the chain is truncated from its ends. With the exception of E, only the entire original polypeptide chain is shown but the closure procedure is repeated as the chain gets truncated, as shown in E.

1. Millett KC, Rawdon EJ, Stasiak A, Sulkowska JI. Identifying knots in proteins. Biochem Soc Trans. 2013;41:533-7
2. Rawdon EJ, Millett KC, Sulkowska JI, Stasiak A. Knot localization in proteins. Biochem Soc Trans. 2013;41:538-41.
3. Tubiana L, Orlandini E, Micheletti C. Probing the Entanglement and Locating Knots in Ring Polymers: A Comparative Study of Different Arc Closure Schemes. Progress of Theoretical Physics Supplement. 2011;(191):192-204.
4. Virnau P, Mirny LA, Kardar M. Intricate knots in proteins: Function and evolution. PLoS Comput Biol 2006;2:e122.
5. Mansfield, ML. Fit to be tied. Nat. Struct. Mol. Biol. 1997;4:166-167.
